# Evaluation of two portable pupillometers to assess clinical utility

**DOI:** 10.2217/cnc-2020-0016

**Published:** 2020-10-28

**Authors:** Rachel Eshima McKay, Michael A Kohn, Elliot S Schwartz, Merlin D Larson

**Affiliations:** 1 Department of Anesthesia & Perioperative Care, Professor, University of California San Francisco, CA 94143, USA; 2 Department of Epidemiology & Biostatistics, Professor, University of California San Francisco, CA 94143, USA; 3 Department of Anesthesia & Perioperative Care, Resident in Anesthesia, University of California San Francisco, CA 94143, USA; 4 Department of Anesthesia & Perioperative Care, Professor Emeritus, University of California San Francisco, CA 94143, USA

**Keywords:** concussion, eye, pupil, pupillary light reflex, pupillometry

## Abstract

**Background:**

Pupillometers have been proposed as clinical assessment tools. We compared two pupillometers to assess measurement agreement.

**Materials & methods:**

We enrolled 30 subjects and simultaneously measured the pupil diameter and light reflex amplitude with an iPhone pupillometer and a portable infrared pupillometer. We then enrolled 40 additional subjects and made serial measurements with each device.

**Results:**

Failure occurred in 30% of attempts made with the iPhone pupillometer compared with 4% of attempts made with the infrared pupillometer (Fisher’s exact p = 0.0001). Method comparison of the two devices used simultaneously showed significant disagreement in dynamic measurements.

**Conclusion:**

The iPhone pupillometer had poor repeatability and suggests that it is not a practical tool to support clinical decisions.

Assessment of the pupillary light reflex (PLR) following a suspected concussion injury is an essential component of the physical examination [1]. Within the past 30 years, portable pupillometers have become available that can analyze the PLR waveform [2–5]. These instruments are designed to measure latencies (ms), constriction velocities (const-vel, mm/s) and reflex amplitudes (RA, mm) from changes in the pupillary diameter (PD; mm) that follow a brief flash of visible light. Many authors agree that these portable instruments provide objective measures from the light reflex with greater accuracy and prognostic value compared with the subjective assessments obtained using the traditional pen light examination [1,2,4,6,7]. As a result, portable pupillometers have been used to study the changes in pupil size and PLR after concussion [1,4,6,7].

Because portable infrared pupillometers are not widely available, several entrepreneurs have proposed that the smartphone can be adapted for use as a portable pupillometer [2,8]. Measuring the PLR with a smartphone would provide a low cost and convenient alternative to the more expensive infrared-based pupillometer. These developers claim that smartphone-based pupillary assessment can be used for a variety of purposes, including tracking the progression and severity of traumatic brain injury and assessing athletes for signs of concussion.

While there are several smartphone-based pupillometers in development, one such application, BrightLamp^®^ (BL; Brightlamp Inc., IN, USA) [8], is currently available for a monthly fee of $39 after a free 3-month trial period. We compared measurements obtained with the BL smartphone-based pupillometer against those of an infrared portable device that has been tested and validated for accuracy, the NeurOptics PLR-3000 (NO; NeurOptics, CA, USA) [9].

With precise, validated desktop pupillometers, const-vel and dilation velocity (dilat-vel) are known to be correlated to RA [10,11]. Any smartphone pupillometer capable of reliably reproducing these relationships would have potential as a valuable tool for gathering timely and important clinical information following concussion injuries. We hypothesized that both instruments would reliably document const-vel and dilat-vel, record reproducibly consistent pupillary dynamic waveforms on first and second trials with a coefficient of variation ≤20%, and exhibit limits of agreement during simultaneous light reflex measurements not exceeding a 20% difference in RA and PD.

## Materials & methods

The study protocol was approved by the Copernicus Institutional Review Board (#1272484), and written consent was obtained from all subjects. The data were collected in December 2019. 70 adult volunteers were recruited and studied at the University of California, San Francisco (CA, USA) presurgical assessment clinic. Volunteers included ambulatory patients, visitors in the clinic waiting area and hospital employees. Exclusion criteria included blindness in one or both eyes, glaucoma or concomitant use of topical ophthalmic medications. Categorical demographic data, including eye color and age range (18–29, 30–49, 50–69 and ≥70 years) were recorded. Size and PLR measurements were obtained with the BL app on the smartphone and the NO according to the instruction manuals for both instruments. All measurements were made by the same investigator (ES Schwartz), who practiced with both instruments for several days prior to enrolling subjects. Upon completion of the measurements, all participants were given a local coffee shop $10 gift card. The BL application was used with the iPhone 8 set in Flash mode, with a measurement duration of 3 s. The iPhone was held in a steady position, with the arm of the investigator resting on a firm table. Room light was approximately 350 lux. At least 1 min separated repeated measurements.

In the first 30 subjects, simultaneous measurements were made to determine agreement between the RA and pupil size measurements obtained with each device. We chose to enlist 30 subjects in order to construct a Bland–Altman plot with at least 40 data points. With the subject seated and facing the investigator, the subject aligned the right eye with a traditional infrared pupillometer and the left eye with the mobile phone camera. The infrared pupillometer was set to have no light flash or background light. The rubber cup of the infrared pupillometer was positioned to exclude ambient light. The flash of light lasting 0.1 s at approximately 1000 lux was initiated from the iPhone, while both instruments simultaneously recorded the light reflex. We attempted to secure two simultaneous measurements of RA and PD on these first 30 subjects. If the device registered measurement failure at any time, we made one additional attempt, but we did not exceed three trials in any subject. The const-vel, dilat-vel and latencies were not recorded while taking simultaneous measurements.

After 30 subjects had been studied with the above protocol, another group of 40 subjects underwent light reflex measurement in the left eye only with each pupillometer in sequence. These sequential measurements were used to determine consistency of repeated measurements with NO and BL, and to establish whether measurements from both instruments exhibited the expected relationship between RA (amplitude) and const-vel (velocity). The trending of const-vel is of prognostic importance after concussion, and absence of any relationship between const-vel and RA in either instrument would indicate error of significant magnitude to preclude its use as a diagnostic tool. We alternated the sequential measurements between the two instruments and sought two sequential measurements of RA, PD, const-vel, dilat-vel and latency from each device. We made one additional measurement attempt if either device registered failure but did not pursue additional measurements if two satisfactory sequential measurements from each device could not be secured after a total of six attempts. We recorded incidence of measurement failure for each device under both protocols. For both instruments the light flash was set at 0.1 s at approximately 1000 lux and the opposite eye was closed and covered by the subject’s hand. We reviewed each BL measurement to ensure that the pupil and iris were positioned within the video recording. All data were loaded into Excel spreadsheets and the measurements were plotted out with time on the x-axis and pupil diameter on the y-axis.

### Statistics

Failure rate of each device was compared using two-sided Fisher’s exact test for simultaneous and sequential measurements. Differences between simultaneous RA and PD measurements were calculated. Standard Bland–Altman plots showed the difference versus average of the two simultaneous measurements. The bias (mean difference), limits of agreement (mean difference ± [1.96*SD]) and the range spanning the upper and lower 95% limits of agreement (minimum detectable change were determined [12]. We considered the maximum acceptable limits of agreement to fall within ≤20% difference between the NO versus BL. For sequential measures, we calculated the repeatability (2.77 × SD) and the within-subject coefficient of variation. The difference between two measurements for the same subject is expected to be less than the repeatability for 95% of pairs of observations. The coefficient of variation represents the variability of repeated measurements as a proportion of their mean. We expected that the coefficient of variation would not exceed 20% in any parameter [13,14]. Paired t-tests were used to compare mean absolute relative differences between the two sequential measurements made with each of the two pupillometers. The absolute relative difference between two measurements is: ([Measurement 2 - Measurement 1]/[Measurement 1 + Measurement 2]/2). Stata version 16 was used for all statistical calculations.

## Results

The age of the subjects was 47 ± 14 years. Eye color was blue, green or hazel in 21 and brown in 49 subjects.

Measurement failure, in which the device could not register any reading, occurred more often with the BL compared with the NO instrument. In the 30 subjects undergoing simultaneous measurements, the BL instrument failed to record a valid scan in six subjects, while the NO device failed in one subject (relative risk = 6.0 [0.8–46.9] for failure with BL vs NO, two-sided Fisher’s exact p = 0.1028). In the 40 subjects undergoing sequential measurements, the BL failed to record the complete parameters of the light reflex in 15 subjects while the NO failed in two subjects (relative risk = 7.5 [1.8–30.7] for failure with the BL vs NO, 2-sided Fisher exact p = 0.0007), and overall the failure rates were 30 versus 3% (relative risk 7.0 [2.2–22.4], 2-sided Fisher exact p = 0.0001). With both devices, all failed measurements occurred in subjects with a brown-colored iris.

### Simultaneous measurements

**Figure 1.**
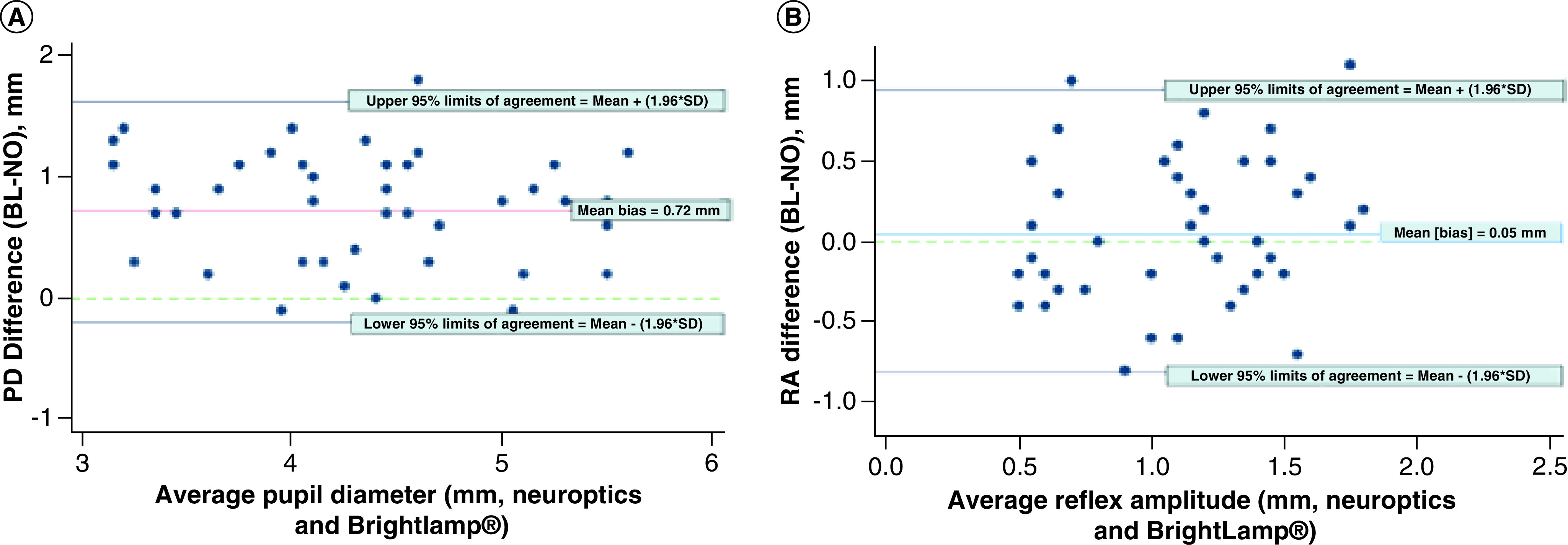
Bland–Altman plots demonstrating disagreements between the two pupillometers. **(A)** Difference versus average of simultaneously measured PD using the NO and BL devices. Upper 95% limit of agreement = 1.6305 and lower 95% limit of agreement = -0.1957, with range of agreement = 1.8262 mm. **(B)** Difference versus average of simultaneously measured RA using the NO and BL devices. Upper 95% limit of agreement = 0.9515, and lower 95% limit of agreement = -0.8472, with range of agreement = 1.7987 mm. BL: BrightLamp^®^; NO: NeurOptic; PD: Pupil diameter; RA: Reflex amplitude.

In 46 simultaneous measurements from 23 subjects, the BL measurement of PD averaged 0.72 mm with upper and lower limits of agreement of 1.63 and -0.20 mm respectively (Figure 1A). The mean difference between RA measured by the BL versus NO was just 0.05 mm, but the Bland–Altman plot for these measurements showed wide scatter around the mean RA difference (Figure 1B), with upper and lower limits of agreement of 0.95 and -0.85. The minimum detectable change (1.80 [1.56–2.04] mm) exceeded the average observed RA measurement from the two devices.

### Sequential measurements

We calculated the percent difference in sequential measurements taken with both instruments in all of the parameters (Table 1). None of these differences exceeded 15% with the NO device, but disagreement was greater than 15% for all sequential parameters except pupil diameter in the BL (Table 1). Assuming no change in the conditions, the difference between a first and subsequent (repeated) measurement should not exceed the repeatability coefficient (2.77 * Sw) in 95% of attempts (Sw = within-subject standard deviation [SD], the square root of the variance). In the BL, the repeatability exceeded ranges spanning clinically relevant changes. Compared with the NO, the BL’s repeatability was one SD higher for PD, three SDs higher for RA, latency and const-vel, and five SDs higher for dilat-vel (Table 1).

**Table 1.** Repeated measurements with each device, showing the average difference, repeatability and within subject coefficient of variation.

Parameter	NeurOptics	BrightLamp^®^	p-value^†^
**Pupil diameter**			
Average; mm	5.12	5.11	
Repeatability; mm (SD)	0.72 (0.26)	0.99 (0.36)	
Coef of variation; % (95% CI)	5.4 (2.8–7.2)	7.0 (5.0–8.6)	
MARD (SD)	**5.0 (5.5)**	**6.9 (6.1)**	**0.3327**
**Reflex amplitude**			
Average; mm/s	1.52	1.15	
Repeatability; mm/s (SD)	0.26 (0.10)	0.58 (0.22)	
Coef of variation; % (95% CI)	6.8 (5.3–8.0)	18.7 (11.3–23.9)	
MARD (SD)	**7.6 (5.4)**	**17.3 (13.1)**	**0.0056**
**Latency**			
Average; s	0.23	0.18	
Repeatability; s (SD)	0.05 (0.02)	0.11 (0.04)	
Coef of variation; % (95% CI)	8.4 (6.1–10.2)	23.5 (18.2–27.8)	
MARD (SD)	**7.4 (8.6)**	**25.8 (24.8)**	**0.0024**
**Constriction velocity**			
Average; mm/s	3.59	1.57	
Repeatability; mm/s (SD)	0.86 (0.31)	1.97 (0.71)	
Coef of variation; % (95% CI)	9.8 (5.0–12.9)	44.2 (32.6– 53.4)	
MARD (SD)	**9.0 (8.7)**	**53.9 (83.4)**	**0.0198**
**Dilatation velocity**			
Average; mm/s	1.23	0.55	
Repeatability; mm/s (SD)	0.37 (0.14)	1.10 (0.41)	
Coef of variation; % (95% CI)	10.3 (7.6–12.4)	52.0 (36.3–64.0)	
MARD (SD)	**11.0 (9.0)**	**82.6 (194.0)**	**0.0902**

† Paired t*-*tests comparing percent change for NeurOptics vs BrightLamp^®^. Statistical analysis was performed on MARD – that parameter is shown in bold.

Repeatability = 2.77 * S_w_, where S_w_ = within subject SD.

95% of differences between first and second measurements will be ≤ the repeatability, assuming no change occurs in conditions affecting the measurements between the attempts [13].

CVw = SD/mean, expressing the random error proportionally to the magnitude of the measurement. We hypothesized that repeated measurements in each instrument should disagree no more than 20%.

MARD between first and second measurements were calculated for each device, and results were compared by two-tailed paired t-tests. See statistical methods for calculation of MARD.

CVw: Coefficient of variation; MARD: Mean absolute relative difference; SD: Standard deviation.

**Figure 2.**
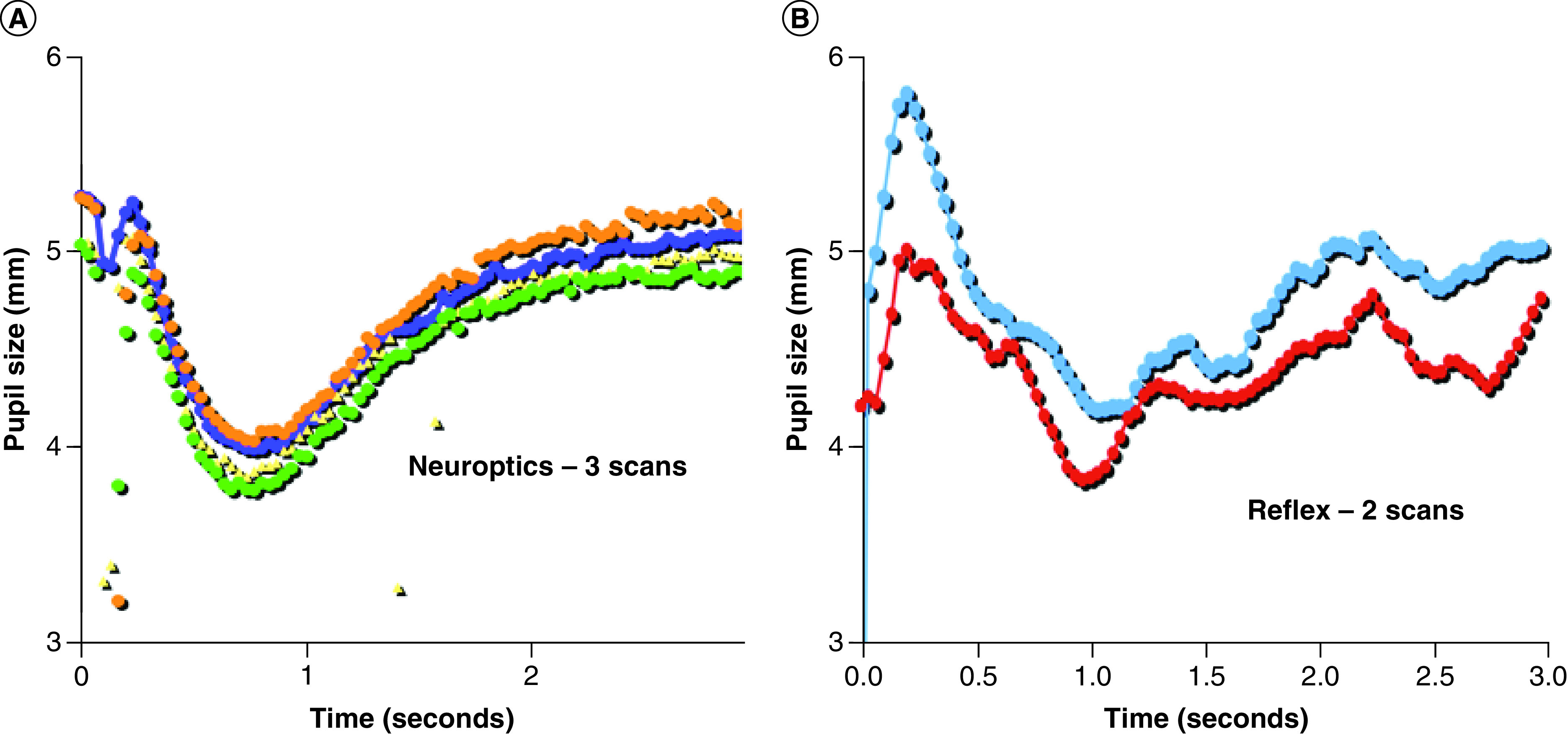
Sequential measurements with the two tested pupillometers. **(A)** NO showing three sequential measurements in the same individual under identical conditions. **(B)** BL showing two sequential light reflex measurements on the same subject under identical conditions. BL: BrightLamp^®^; NO: NeurOptic.

**Figure 3.**
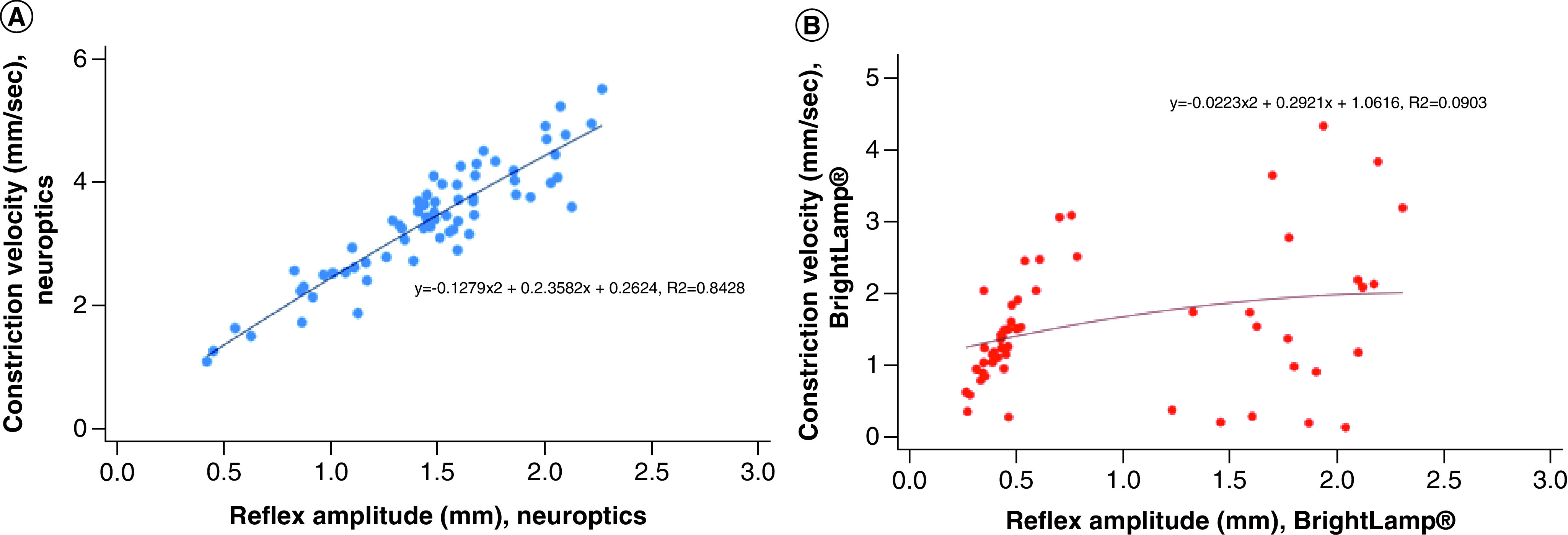
Relationship between RA and const-vel with the two instruments. **(A)** The relationship between RA and const-vel with the NO infrared pupillometer. This correlation is similar to that reported using high precision desktop pupillometers. **(B)** No significant relationship between RA and const-vel was observed with the BL smartphone-based device. BL: BrightLamp^®^; const-vel: Constriction velocity; NO: NeurOptic; RA: Reflex amplitude.

Sequential measurements for const-vel and dilat-vel showed significant correlation using the NO instrument but no significant correlation using the BL (Table 1). Significant irregularities were observed between the two sequential measurements using the BL pupillometer (Figure 2B) whereas the NO measurements were nearly identical (Figure 2A). The relationship between RA and const-vel was highly significant (y = -0.1279x^2^ + 2.3582x + 0.2624; R^2^ = 0.8428, p < 0. 001) when measurements were taken with the NO pupillometer (Figure 3A). Likewise, the relationship between RA and dilat-vel was significant (p < 0.001) (not shown). Those relationships could not be established with the BL, as the correlation coefficients were both below 0.1 (Figure 3B).

## Discussion

We evaluated two pupillometers that have been promoted to assess the PLR waveform after concussion. With simultaneous measurements the two devices did not record the same RA. Our results indicate that the NO would reproduce the parameters of the light reflex but the waveform with the smartphone-based device was unreliable and could not reproduce the relationships between const-vel, dilat-vel and RA that have been demonstrated in healthy subjects [10,11]. The smartphone pupillometer (BL) produced sequential measurements that differed to an extent that would render it unacceptable for clinical decision-making (Table 1 & Figure 2B).

Although modest variability in sequentially measured light reflexes can be observed within normal individuals, closely timed repeated light reflexes have remarkably similar waveforms in alert healthy subjects [15]. Our measurements were taken without changes in ambient light, distracting noise or cognitive challenges that are known to alter light RA. Some degree of consistency must be present in sequential measurements, because after concussion injuries the trends of light reflex data might be of greater value than any one isolated measurement. Repeat measurements with the NO pupillometer were within 11% of the initial values in each individual, but difference in sequential measurements with the BL ranged between 15 and 83%. To put the importance of precision in context, changes in const-vel, dilat-vel and latency have been reported to be within a range of 13–22% following blast injury [7].

During the simultaneous measurements, we recorded with the smartphone from one eye and the NO device with the other eye, with the stimulating light flash generated from the smartphone camera. As direct and consensual reflexes in humans are the same [15], the recorded reflexes should also be the same. However, the two simultaneous measurements differed substantially, thus confirming our conclusion that the smartphone-based pupillometer cannot be used for patient care or triage purposes, including assessment of suspected concussion.

Previous investigators have focused on const-vel or dilat-vel as objective measures to follow after concussion injury [1,6]. Other studies have established that const-vel is related to the RA, and RA is in turn related to the size of the pupil [10,11]. Our data confirm those relationships with the NO device, but not with the BL. Of additional interest, we observed that the BL pupillometer was rendering latencies that were shorter than have been reported by high precision pupillometers [16].

We did note that sequential measurements of PD were moderately consistent with both devices. Although severe traumatic brain injury can dilate the pupil from compression of the third cranial nerve, PD alone is unlikely to be an adequately sensitive indicator early in the course of injury, or following mild concussions [1].

It is possible that the smartphone-based device might be more accurate when used under different lighting conditions. We designed the study to reproduce a typical level of indoor lighting, with the subjects not looking directly into a light source. Detection of the pupillary margin is known to be more difficult in subjects with a brown iris when using visible compared with infrared light and that observation has been a driving factor behind the development of infrared pupillometers. We speculate that increasing the direct illumination of the eye during the measurement might improve the consistency of the smartphone pupillometer, but that maneuver would also constrict the pupil and result in altered values of const-vel and RA.

A limitation of our study is that we did not measure the light reflex with either device in concussed subjects. Others have investigated the changes in the PLR after traumatic head injuries but whether either instrument would provide valuable diagnostic information after concussion was not addressed by our study. In addition, 70% of our subjects had brown eyes; although this might be a limitation of our study, any instrument that is promoted to measure the parameters of the light reflex should be capable of providing accurate measurements, whatever the eye color. It is estimated that 79% of the world’s population has brown eyes (World Atlas).

## Conclusion

We evaluated two portable pupillometers that have been promoted to document the characteristics of the PLR following concussion injuries. The infrared portable instrument rendered consistent sequential values, but the smartphone-based instrument did not exhibit reliable recordings and would not be an acceptable device to use as a clinical tool to track the severity of concussion after head injury.

## Future perspective

Smartphone-based pupillometry will be promoted in the near future. A valid assessment of each new device by unbiased investigators should be a prerequisite before these instruments can be recommended for clinical decision making.

**Executive summary**

- Analysis of the pupillary light reflex has been suggested as a useful measure to document after suspected concussion injuries.
- Accurate portable instruments are recommended for ease of use and convenience.
- Infrared portable pupillometers have been in use for over 20 years and have been tested for accuracy.
- We compared the accuracy of an infrared portable pupillometer to a smartphone-based pupillometer designed to measure the dynamic characteristics of the pupillary light reflex.
- The two devices did not document the same reflex when used simultaneously.
- The smartphone-based pupillometer did not record consistent repeat measurements.
- The smartphone-based pupillometer is a novel idea that records the light reflex but is not precise and cannot be recommended to monitor the light reflex after concussion injuries.
